# Modeling and simulation of an anatomy teaching system

**DOI:** 10.1186/s42492-019-0019-4

**Published:** 2019-08-02

**Authors:** Xiaoqin Zhang, Jingyi Yang, Na Chen, Shaoxiang Zhang, Yifa Xu, Liwen Tan

**Affiliations:** 1 Institute of Digital Medicine, School of Biomedical Engineering and Medical Imaging, Army Medical University (Third Military Medical University), Chongqing, 400038 China; 2 Shandong Digihuman Technology Co., Inc, Jinan, 250101 China

**Keywords:** Chinese visible human, Anatomy knowledge ontology, Virtual reality, Anatomy teaching

## Abstract

Specimen observation and dissection have been regarded as the best approach to teach anatomy, but due to the severe lack of anatomical specimens in recent years, the quality of anatomy teaching has been seriously affected. In order to disseminate anatomical knowledge effectively under such circumstances, this study discusses three key factors (modeling, perception, and interaction) involved in constructing virtual anatomy teaching systems in detail. To ensure the authenticity, integrity, and accuracy of modeling, detailed three-dimensional (3D) digital anatomical models are constructed using multi-scale data, such as the Chinese Visible Human dataset, clinical imaging data, tissue sections, and other sources. The anatomical knowledge ontology is built according to the needs of the particular teaching purposes. Various kinds of anatomical knowledge and 3D digital anatomical models are organically combined to construct virtual anatomy teaching system by means of virtual reality equipment and technology. The perception of knowledge is realized by the Yi Chuang Digital Human Anatomy Teaching System that we have created. The virtual interaction mode, which is similar to actual anatomical specimen observation and dissection, can enhance the transmissibility of anatomical knowledge. This virtual anatomy teaching system captures the three key factors. It can provide realistic and reusable teaching resources, expand the new medical education model, and effectively improve the quality of anatomy teaching.

**Electronic supplementary material:**

The online version of this article (10.1186/s42492-019-0019-4) contains supplementary material, which is available to authorized users.

## Introduction

Anatomy is one of the core courses in medical education, and it is a compulsory course for every medical student and for students in fields related to medicine. Anatomy is a morphological discipline that requires a large amount of human morphological material as a bridge for knowledge dissemination. Traditional anatomy teaching uses wall charts, books, slides, anatomical specimens, and practical anatomy as teaching resources and methods [1–3]. The use of physical anatomical specimens and practical anatomy can obtain the best teaching results [4, 5].

With the expansion of medical education and the reduction in human anatomical specimens [6], as well as the limitations of time and place for anatomical training, the quality of anatomy teaching has been seriously affected [7, 8]. Given the needs of anatomy teaching, the use of virtual reality (VR) technology to construct a virtual anatomy teaching system, based on the Chinese Visible Human (CVH) dataset [9, 10], could therefore provide real and reusable teaching resources for anatomy teaching [11–13]. In addition to traditional teaching functions, virtual anatomy teaching systems have the advantages of multi-level, multi-angle specimen observation and non-destructive virtual anatomy [14], among others. This relieves the pressure on anatomical teaching. To a certain extent, it can replace traditional anatomy teaching.

### A virtual anatomy teaching system

**Fig. 1:**
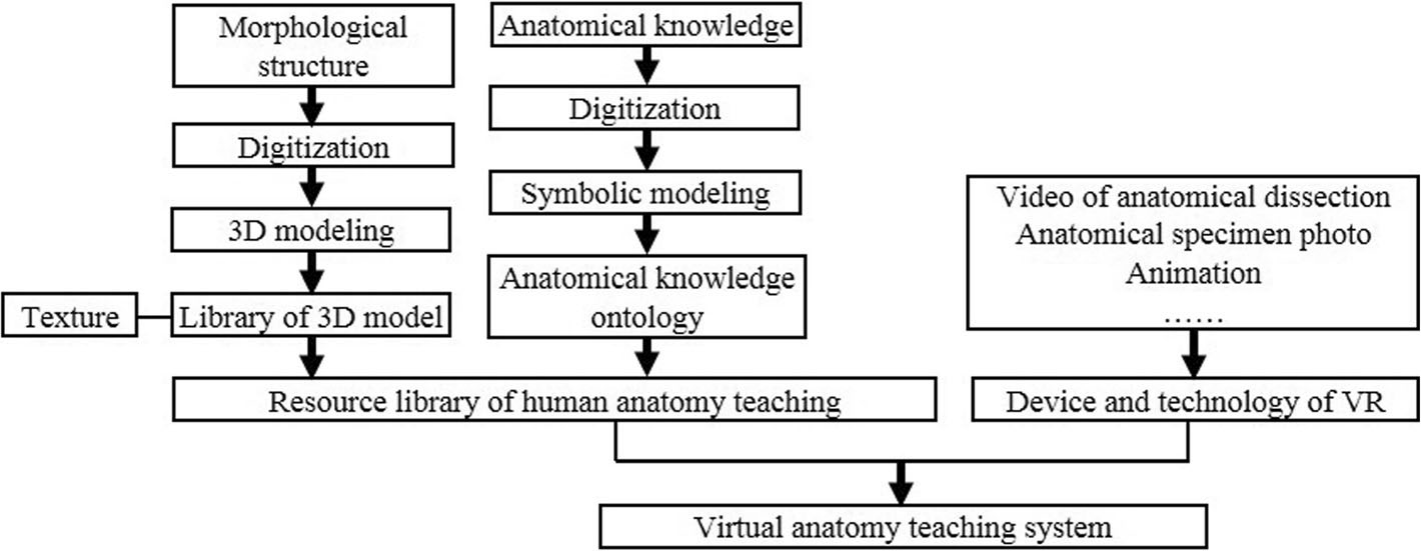
Basic steps in the development of a virtual anatomy system

A complete virtual anatomy teaching system includes a variety of anatomical teaching materials, such as a human morphological three-dimensional (3D) model, an anatomical knowledge ontology, an anatomical specimen map, and a video of anatomical operations. The design of a virtual simulation system would be constructed according to the needs of the teaching objectives. The basic steps in the development of a virtual anatomy system are shown in Fig. 1.

The development of a virtual anatomy teaching system includes the following aspects: (1) digitization and modeling of human morphological structure to construct a 3D model library with texture; (2) digitization of anatomical knowledge and modeling symbols to construct an anatomical knowledge ontology; (3) semantic association of the 3D model library, anatomical knowledge ontology, and conventional anatomical resources (such as videos, maps, animations, etc.) to construct a resource library of human anatomy teaching; and (4) construction of a virtual anatomy teaching system appropriate for the particular teaching purpose using VR devices and technology.

Therefore, the virtual simulation of anatomical teaching systems must address three core issues: modeling, perception, and interaction. Modeling refers to the kind of model to be built and the construction of specific models to meet the needs of virtual simulation; perception refers to the kind of knowledge and experience that needs to be communicated by the system, as well as how they can be effectively perceived by the learner and whether the perceived content is required by the learner; interaction refers to ensuring that the form of interaction with the system is an effective means of knowledge dissemination.

### Modeling

Virtual simulation models are the core and foundation of the operation of virtual anatomy teaching systems, especially 3D digital anatomical models. A virtual simulation teaching system with only digital anatomical models can only transmit knowledge of morphology. It cannot express the anatomical knowledge refined by predecessors and the related physiological functions. The simulation models of the virtual anatomy teaching system constructed here contain 3D digital anatomical models of human morphological structure and anatomical knowledge ontology.

#### Morphological modeling

The CVH images are optical images obtained by milling and cutting human cadaver specimens using frozen tomographic milling technology. These images have ultra-high spatial resolution, and the structures are recognizable. Complete and realistic 3D models, that is, digital anatomical specimens, can be obtained by image segmentation and 3D reconstruction of these images. Most of the virtual simulation models used in the virtual anatomy teaching system come directly from these digital anatomical specimens.

In order to express the authenticity and comprehensibility of specimens more effectively, some 3D reconstructed models usually need to be patched and beautified, such as 3D models of the brain and sulcus. Some structures lacking original data sources need to be hand drawn according to their morphological features and spatial adjacency, such as white matter fiber bundles of the brain, blood vessels of the fingers, and diaphragms. Obvious feature information of some anatomical structures is difficult to obtain using optical imaging tomography, usually requiring additional imaging data for supplementation, such as clinical imaging, tissue sectioning, and other data. For example, the white matter fiber bundle of the brain is white on the sectional specimen, so it is impossible to distinguish each white matter fiber bundle. It is necessary to use the diffusion tensor imaging data of white matter to construct each white matter fiber bundle required for the virtual simulation teaching system. In addition, morphological information of each nerve nucleus in the brain stem needs to be obtained by stained tissue sections, and the dynamic 3D model of the heart comes from the sequence data of the multi-period in the cardiac physiological cycle.

**Fig. 2:**
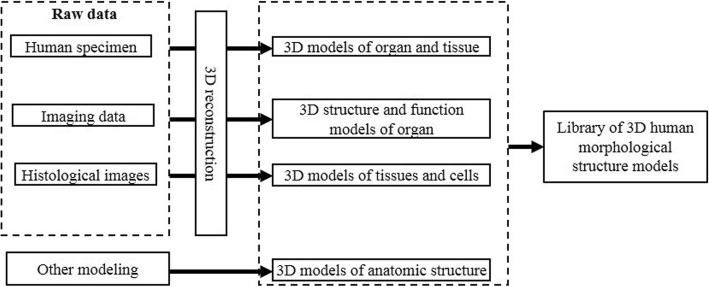
Modeling process of human morphological structures

Under the principle of not affecting the morphological expression of the structure, the number of patches of the constructed 3D models is deleted as appropriate, and pseudo-color and texture mapping processing is performed. Finally, a 3D model library of human morphological structures required by the virtual simulation teaching system is formed (Fig. 2). The selected texture map is based on the colors and textures of the real anatomical specimens and combines the functional expressions of the human body structure. Learners can thereby obtain visual and intuitive perceptions from the 3D digital anatomical models. For example, the map of the muscle model is based on the actual anatomical specimen map, and the red arterial blood vessels indicate the presence of oxygen-rich arterial blood.

#### Knowledge modeling

First, it is necessary to extract anatomical knowledge from anatomical textbooks, as well as the correct anatomical nouns, according to the proposed rules and to formulate the anatomical terminology and its characteristic extraction and knowledge derivation rules according to the characteristics of anatomical knowledge. It is then necessary to construct the anatomical knowledge ontology, Bo = {At, Rel, P, Rol, Mk}, where Bo is the anatomical ontology, At is the anatomical terms, Rel is the relationship between the terms, P is the attributes of the anatomical nouns, Rol is the rules and functions related to the anatomical nouns, and Mk is the metadata providing clear specifications of the attributes, relationships, rules, functions, and definitions.

**Fig. 3:**
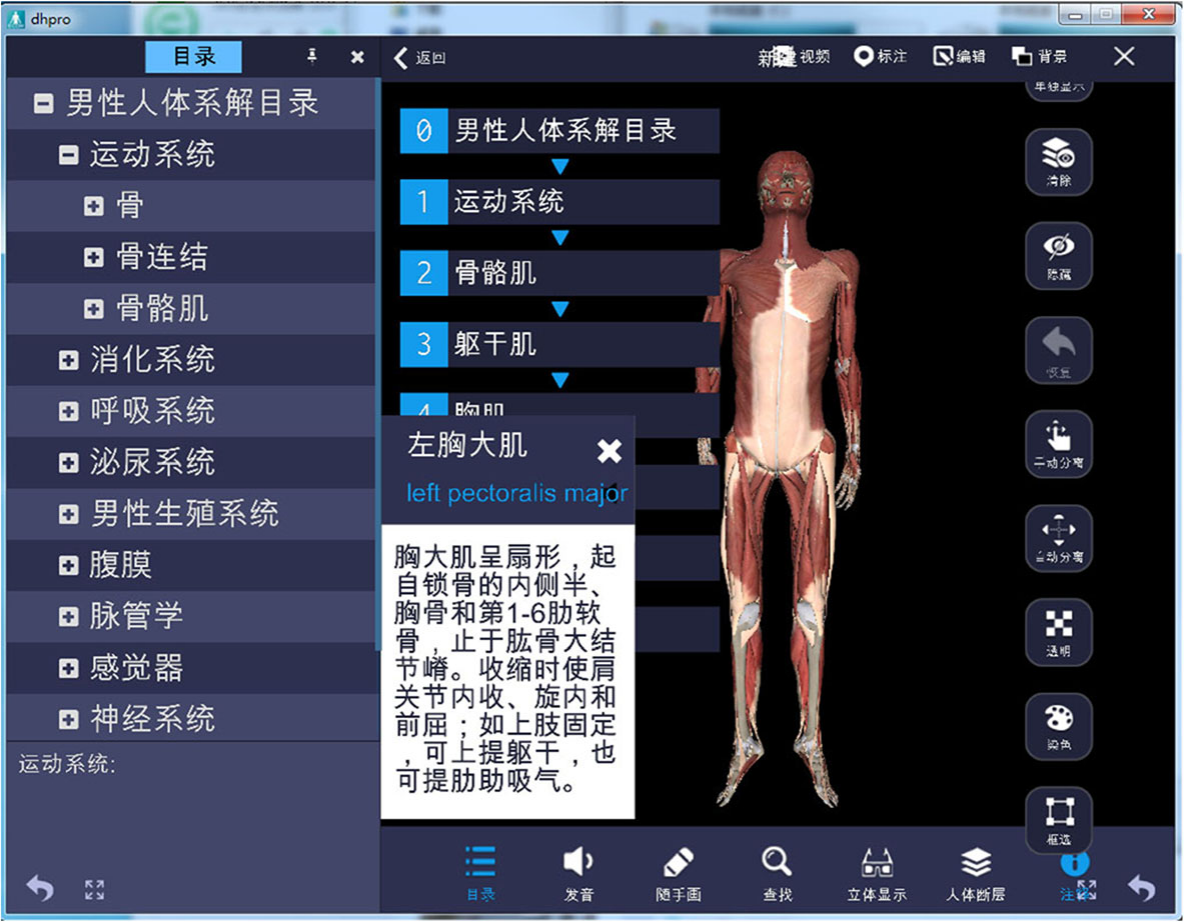
Example of knowledge modeling

Anatomy knowledge ontology is an abstraction of anatomical knowledge, covering the anatomical knowledge points that medical students need. It includes anatomical nouns, morphological descriptions, physiological functions, and other related information but lacks an intuitive morphological display. Assigning the ontology to a specific individual can realize the instantiation of anatomy knowledge ontology, Bs = {Bo, Bi, Bm, Ps, RL}, where Bo is the anatomical ontology, Bi is the basic information of the individual, Bm is the 3D model of the individual, Ps is the individual tomographic image, and RL is the relationship between spatial position and terminology. The models instantiated inherit all the relationships and attributes in the anatomy ontology and contain the unique information and 3D visualization model (Fig. 3). The instantiation of the anatomical ontology can realize the organic relationship between the spatial information and the anatomical knowledge of the morphological structure, which is conducive to the perception and propagation of anatomical knowledge.

Virtual anatomical models are the basis of the virtual anatomy teaching system. The advantages and disadvantages of the simulation models directly affect the perceived effects and the convenience of interactions. The accuracy and integrity of the models determine the accuracy and integrity of knowledge expression, which plays a vital role in the development of the simulation system.

### Perception

Knowledge perception is intended to help students acquire knowledge. It is necessary to consider the integrity, systematization, and authenticity of the knowledge, as well as whether its performance is realistic, whether the sense of operation is close to reality, and whether it is readily acceptable.

**Fig. 4:**
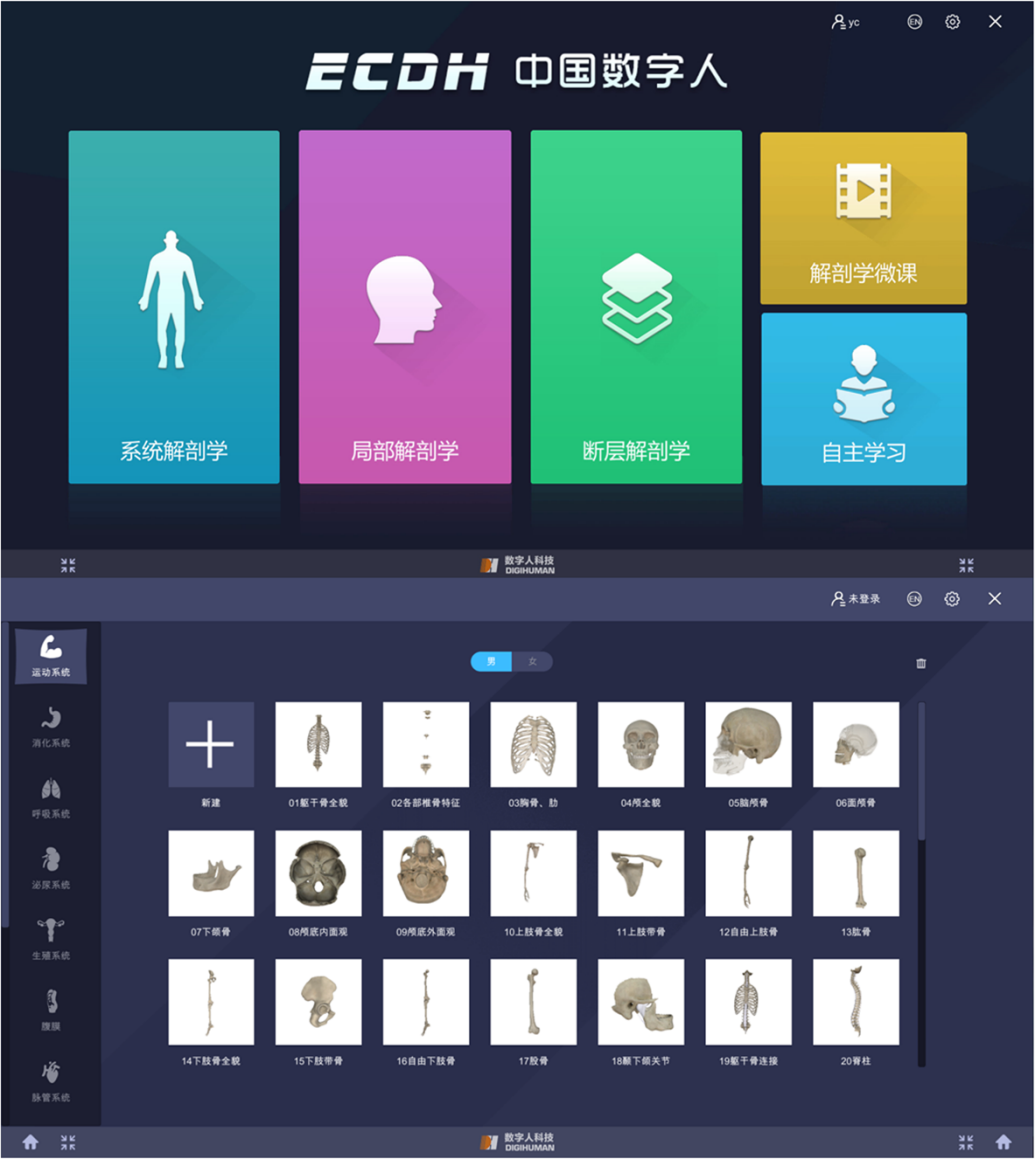
Yi Chuang digital human anatomy teaching system

**Fig. 5:**
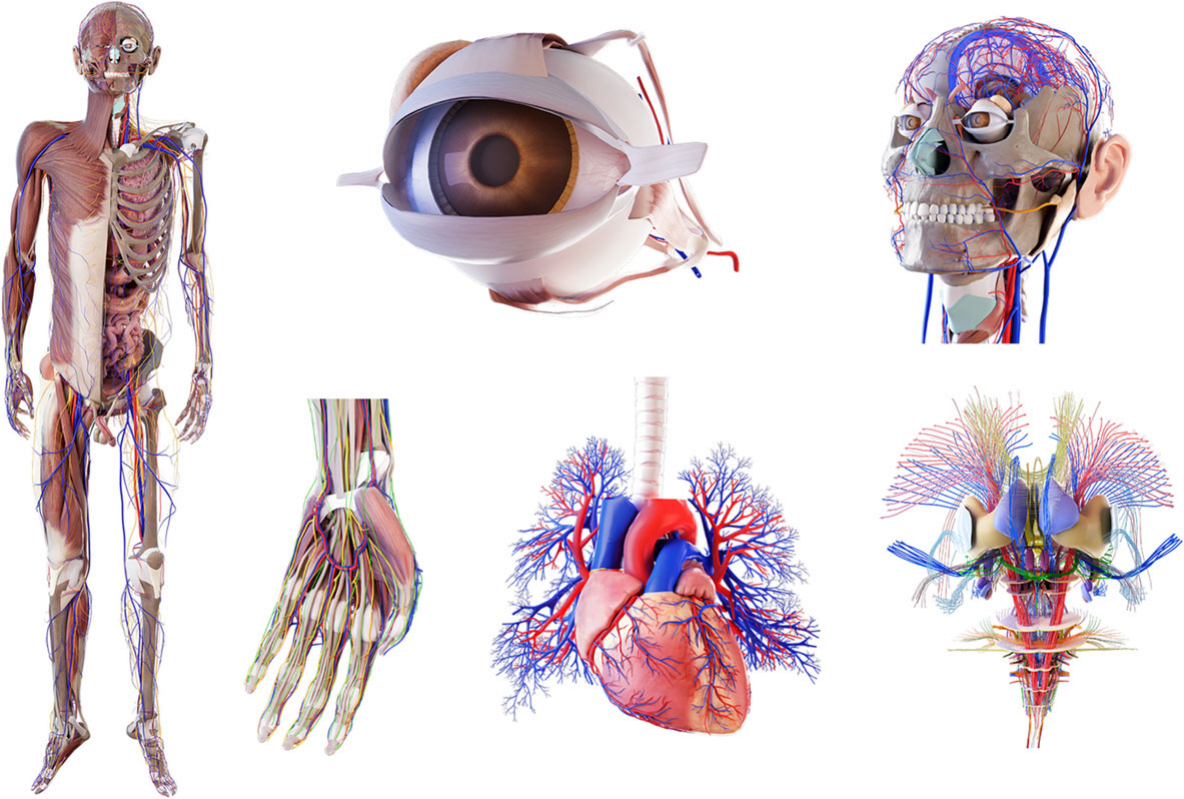
Examples of the virtual anatomical models

The Yi Chuang Digital Human Anatomy Teaching System (Fig. 4) is a virtual anatomy teaching system built on the basis of digital human data from China. There are 5000 digital models reconstructed by 3D modeling and manual drawing, meeting the teaching requirements of medical undergraduate and postgraduate stages. The constructed 3D models (Fig. 5) are meticulous and distinctive, capable of truly reflecting the morphological features of normal human anatomical structures and spatial proximity relationships. The use of the appropriate texture maps make it possible to interpret the characteristics of anatomical structures in terms of morphological structure and functional information that are authentic and credible in terms of visual perception. For some abstract concepts, the knowledge points that need special identification are supplemented by other materials, such as nerve conduction, joint movement, and practical anatomy operations, which are displayed through animation and video (Additional file 2).

The design of the system’s content fully considers the teaching needs of different levels and different purposes and sets up multiple anatomy courses, including specific professional anatomy courses. Functionally, systematic anatomy, topographic anatomy, sectional anatomy, and surface anatomy are provided, and special professional anatomy courses, such as nursing anatomy and sports anatomy, are distinguished. The knowledge system expressed by the system is complete, systematic, and authentic. The knowledge in the virtual environment is realistic at the visual level and has a long-term visual tolerance.

Anatomy knowledge is the main subject of the virtual anatomy teaching system but also the core content of knowledge communication. This system guarantees a realistic perception of knowledge. The learner acquires the anatomical knowledge through their perception of the system. The effectiveness of the system’s perceivability determines the learner’s efficiency in grasping the knowledge, which reflects the rationality of the system design. The richness of the perceived content determines the integrity and systematization of the system.

### Interaction

The interaction of the system must focus on the transmissibility of knowledge. The selection and design of interactive systems should be based on the habits of learning and dissection, focusing on visual and auditory interactions. The convenience, usability, and sustainability of interaction are key factors in whether a virtual simulation system can be used in practice.

**Fig. 6:**
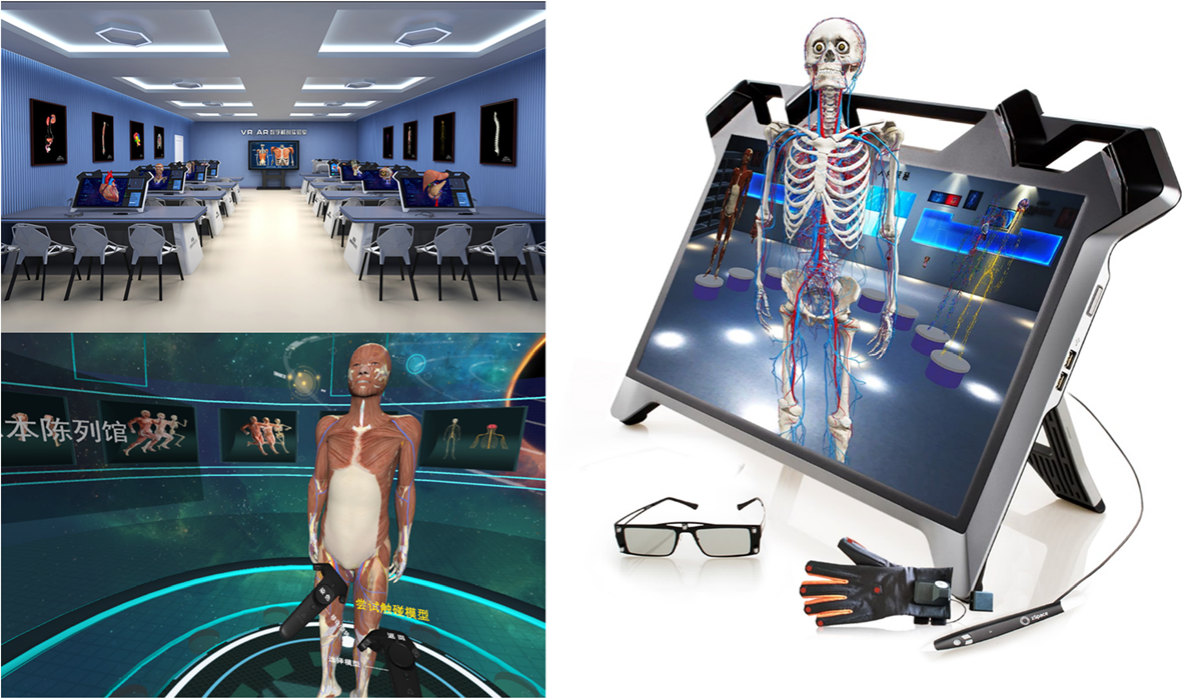
Interaction of the virtual anatomy system

In terms of interactive devices, the virtual anatomy teaching system that we constructed is divided into two types: a 3D plane interactive system and a 3D stereo interactive system. The 3D plane interactive system mainly involves a desktop computer and a touch screen. The 3D stereo interactive system mainly involves a desktop stereo interactive system, a 3D stereoscopic projection system, and a helmet-type stereo interactive system. Different interactive systems have different teaching purposes and effects (Fig. 6) (Additional file 1).

The 3D plane interactive systems generally use a flat panel display and projection as the display interface, with a mouse used as an interactive tool for teaching content. The selection of knowledge content and the virtual anatomical operation of the 3D models through hyperlinks and semantic associations are generally applicable to single-person learning. All the display devices of a 3D stereo interactive system have 3D stereoscopic display functions, and the input devices usually have multiple-degree-of-freedom interactive devices, such as six-dimensional handles, styluses, and data gloves. The desktop stereo interactive system and the helmet-type stereo interactive system are mainly used for personal learning. The 3D stereoscopic projection system is suitable for broadcast teaching, and the helmet-type stereo interactive system is suitable for adding a virtual anatomical environment with a strong sense of presence. Because a stereo interaction system entails a certain amount of visual fatigue and is not suitable for long-term use, it is more suitable for the study of spatial shape knowledge and the adjacent relationships of anatomical structures. The learning of other knowledge points can use the 3D plane interactive system.

In terms of knowledge interaction, the virtual anatomy teaching system uses the word, semantic association, and contrast approaches for quick selection, jumping, and multimode display. The settings of words can be flexible for the teachers’ and students’ learning content, reducing the users’ concerns about the operational steps, allowing them to focus on the acquisition of knowledge. Semantic association can realize the association and jumps between different knowledge system contents (such as systematic anatomy and topographic anatomy), thereby realizing the visualization and rapid positioning of knowledge. An overlay display of a variety of anatomical materials can not only deepen understanding of knowledge but also can guide its learning and mastery.

In terms of the interaction of 3D digital models, the virtual anatomy teaching system implements the display of shallow and deep structures, as well as the relationship between morphological structures, through display, concealment, and transparency. In addition, we can observe the digital 3D morphology of anatomical specimens by rotation, focus on an organ in its entirety, or zoom into local details of morphological features. Virtual cutting of digital human datasets enables the display of any section. The use of specific combinations of models makes it possible to realize the display of local hierarchies and can be compared with dissection to guide students’ operations.

## Discussion

With the help of a variety of interactive devices and technologies, the teaching effect of this virtual anatomy teaching system is close to that of physical specimens. This virtual system can constitute an interactive teaching resource directly used for anatomy teaching, self-study, and dissection. Anatomical specimens required for anatomy teaching can be replaced to some extent by 3D digital anatomical models in this virtual anatomy teaching, not being limited to teaching laboratories [1, 15, 16]. Teaching with a virtual anatomy teaching system has many outstanding advantages over traditional anatomy teaching, such as the non-destructive nature of the specimens, the arbitrary combination of 3D structure displays, and the rapid positioning and association of various forms of anatomical knowledge. These can make up for the shortcomings in current anatomy teaching, enhancing the teaching effect [14, 17].

A particular digital device can only obtain structural data within a finite scale range. Digital information of all required structures is also difficult to obtain through a single imaging method [7]. In this virtual anatomy teaching system, the combined application of multi-scale and multi-modal data ensures the integrity, systematization, and authenticity of the library of 3D digital models [18, 19]. Accurate and complete digital anatomical specimen models are constructed by CVH datasets [14]. These 3D digital models constitute most of the morphological data in the virtual anatomy teaching system. Supplemented by clinical imaging data, tissue section data, and hand-drawn models, it can compensate for the CVH’s lack of spatial scale and single imaging modality. In the authentic expression of the models, the texture map is produced by combining the real picture of the actual specimen with the pseudo-color that can enhance perception, such as the texture and color of the muscles and tendons, and the exaggerated color expression of the blood vessel. This can effectively enhance the student’s anatomical understanding and memory.

Moreover, the construction and instantiation of anatomical knowledge ontology contributes to the semantic association of anatomical terms, concepts, physiological functions, and morphological structures, which enables the organic integration of various kinds of anatomical knowledge, ensuring the integrity and systematization of knowledge expression [20–22].

This virtual anatomy teaching system is diverse in its representation of models. It can quickly select the model and knowledge structure to be displayed and can display both the 3D model and the sections in combination. It can quickly produce an accurate observation of a single model. Supplemented by 3D animations, it can enhance the understanding of anatomical knowledge. It can simulate multi-level observations of anatomical structures in local anatomy, as well as some special virtual anatomical display methods, enhancing the perception of knowledge [23].

The realistic reproduction of 3D digital anatomical models, the organic association of anatomical knowledge, and the use of various interactive means enable this virtual anatomy teaching system to reflect fully the transmissibility and usability of knowledge. These features make the virtual anatomy teaching system similar in perception to actual anatomy teaching and can achieve the effect of actual anatomy teaching. The true, complete, and correct digital anatomical models and anatomical knowledge ontology are the core of anatomical knowledge dissemination. Combining the 3D plane with the 3D stereo display mode, 3D spatial information can be obtained, and long-term knowledge acquisition can be generated. The mouse and multiple-degree-of-freedom interactive device can meet the operating behaviors of the masses to a certain extent and can approach the actions of actual dissection. However, due to the large technical difficulty of digital model cutting and deformation, the force feedback device is still struggling to reproduce the real feel. The virtual anatomy teaching system thus still has a major shortfall in the simulation of practical anatomy.

## Conclusion

The purpose of constructing the virtual anatomy teaching system is to spread anatomical knowledge. The core content is the construction of 3D digital models, the perception of anatomical knowledge, and the interaction of systems and knowledge. In the design and application of virtual anatomy teaching systems, the simulation models are the foundation, the perceived content comprises the main body, and the interaction is the means of knowledge dissemination. The pros and cons of these determine the transmissibility of knowledge and the acceptability of the system. The applicability of the virtual anatomy teaching system depends on the fidelity of the 3D models, the integrity and systematization of the anatomical knowledge, and the interoperability of the system. In particular, effective, convenient, and durable interaction is the key to determining the success or failure of the virtual anatomy teaching system.

## Additional files

Additional file 1: Interaction of the virtual anatomy teaching system. (MP4 1285 kb)

Additional file 2: Perception of knowledge in the system. (GIF 5992 kb)

## Data Availability

The data that support the findings of this study are not publicly available due to copyright, but related information can be accessed by the websites, http://cvh.tmmu.edu.cn and http://www.digihuman.com.

## Abbreviations

3D: Three-dimensional
CVH: Chinese Visible Human
VR: Virtual reality

## References

[1] Keedy Alexander W., Durack Jeremy C., Sandhu Parmbir, Chen Eric M., O'Sullivan Patricia S., Breiman Richard S. (2011). Comparison of traditional methods with 3D computer models in the instruction of hepatobiliary anatomy. Anatomical Sciences Education.

[2] McLachlan John C, Patten Debra (2006). Anatomy teaching: ghosts of the past, present and future. Medical Education.

[3] Brenton Harry, Hernandez Juan, Bello Fernando, Strutton Paul, Purkayastha Sanjay, Firth Tony, Darzi Ara (2007). Using multimedia and Web3D to enhance anatomy teaching. Computers & Education.

[4] McMenamin P. G., McLachlan J., Wilson A., McBride J. M., Pickering J., Evans D. J. R., Winkelmann A. (2018). Do we really need cadavers anymore to learn anatomy in undergraduate medicine?. Medical Teacher.

[5] Chapman Stephen J., Hakeem Abdul R., Marangoni Gabriele, Prasad K.R. (2013). Anatomy in medical education: Perceptions of undergraduate medical students. Annals of Anatomy - Anatomischer Anzeiger.

[6] China MoHo (2011). China health statistics yearbook.

[7] Wu Yi, Luo Na, Tan Li-Wen, Fang Bing-Ji, Li Ying, Xie Bing, Xu Hao-Tong, Hu Nan, Yang Wei-Ping, Wu Wei, Lamers Wouter H., Zhang Shao-Xiang (2012). Comparative study of thin sectional anatomical images from Chinese Visible Human data set and computed tomography images of superior mediastinum. Clinical Anatomy.

[8] Wu Yi, Zhang Shao-Xiang, Luo Na, Qiu Ming-Guo, Tan Li-Wen, Li Qi-Yu, Liu Guang-Jiu, Li Kai (2010). Creation of the digital three-dimensional model of the prostate and its adjacent structures based on Chinese visible human. Surgical and Radiologic Anatomy.

[9] Zhang Shao-Xiang, Heng Pheng-Ann, Liu Zheng-Jin (2006). Chinese visible human project. Clinical Anatomy.

[10] Zhang Shao-Xiang, Heng Pheng-Ann, Liu Zheng-Jin, Tan Li-Wen, Qiu Ming-Guo, Li Qi-Yu, Liao Rong-Xia, Li Kai, Cui Gao-Yu, Guo Yan-Li, Yang Xiao-Ping, Liu Guang-Jiu, Shan Jing-Lu, Liu Ji-Jun, Zhang Wei-Guo, Chen Xian-Hong, Chen Jin-Hua, Wang Jian, Chen Wei, Lu Ming, You Jian, Pang Xue-Li, Xiao Hong, Xie Yong-Ming, Cheng Jack Chun-Yiu (2004). The Chinese Visible Human (CVH) datasets incorporate technical and imaging advances on earlier digital humans. Journal of Anatomy.

[11] Linke R, Leichtle A, Sheikh F, Schmidt C, Frenzel H, Graefe H et al (2013) Assessment of skills using a virtual reality temporal bone surgery simulator. Acta Otorhinolaryngol Ital 33(4):273–281 PMC3773961 24043916

[12] Rosseau Gail, Bailes Julian, del Maestro Rolando, Cabral Anne, Choudhury Nusrat, Comas Olivier, Debergue Patricia, De Luca Gino, Hovdebo Jordan, Jiang Di, Laroche Denis, Neubauer Andre, Pazos Valerie, Thibault Francis, DiRaddo Robert (2013). The Development of a Virtual Simulator for Training Neurosurgeons to Perform and Perfect Endoscopic Endonasal Transsphenoidal Surgery. Neurosurgery.

[13] Teishima Jun, Hattori Minoru, Matsubara Akio (2013). Psychological factor, metacognition, is associated with the advantage of suturing techniques acquired on a virtual reality simulator of robot-assisted surgery. International Journal of Urology.

[14] Fang Binji, Wu Yi, Chu Chun, Li Ying, Luo Na, Liu Kaijun, Tan Liwen, Zhang Shaoxiang (2016). Creation of a Virtual Anatomy System based on Chinese Visible Human data sets. Surgical and Radiologic Anatomy.

[15] Hisley Kenneth C., Anderson Larry D., Smith Stacy E., Kavic Stephen M., Tracy J. Kathleen (2007). Coupled physical and digital cadaver dissection followed by a visual test protocol provides insights into the nature of anatomical knowledge and its evaluation. Anatomical Sciences Education.

[16] Saltarelli Andrew J., Roseth Cary J., Saltarelli William A. (2014). Human cadavers Vs. multimedia simulation: A study of student learning in anatomy. Anatomical Sciences Education.

[17] Houser JJ, Kondrashov P (2018). Gross anatomy education today: the integration of traditional and innovative methodologies. Mo Med.

[18] Nyamse Victor, Charissis Vassilis, Moore J. David, Parker Caroline, Khan Soheeb, Chan Warren (2013). The Design Considerations of a Virtual Reality Application for Heart Anatomy and Pathology Education. Virtual, Augmented and Mixed Reality. Systems and Applications.

[19] James Alex Pappachen, Dasarathy Belur V. (2014). Medical image fusion: A survey of the state of the art. Information Fusion.

[20] de Bono B, Hunter P (2012). Integrating knowledge representation and quantitative modelling in physiology. Biotechnol J.

[21] Pommert Andreas, Höhne Karl Heinz, Pflesser Bernhard, Richter Ernst, Riemer Martin, Schiemann Thomas, Schubert Rainer, Schumacher Udo, Tiede Ulf (2001). Creating a high-resolution spatial/symbolic model of the inner organs based on the Visible Human. Medical Image Analysis.

[22] Cerveri P., Pinciroli F. (2001). Symbolic Representation of Anatomical Knowledge: Concept Classification and Development Strategies. Journal of Biomedical Informatics.

[23] Hamrol Adam, Górski Filip, Grajewski Damian, Zawadzki Przemysław (2013). Virtual 3D Atlas of a Human Body – Development of an Educational Medical Software Application. Procedia Computer Science.

