# Supplementary figures and images for: Modeling and simulation of an anatomy teaching system
Source: Vis Comput Ind Biomed Art. 2019 Aug 2;2:8. doi: 10.1186/s42492-019-0019-4 (PMC7099570; doi:10.1186/s42492-019-0019-4)

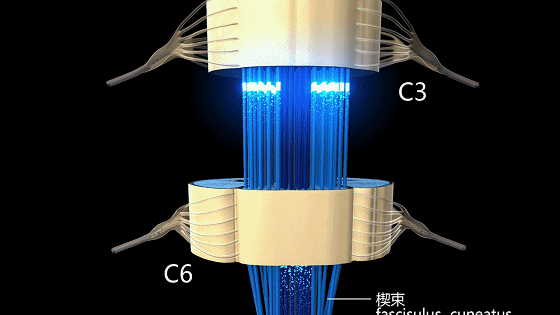

Supplement: Supplementary file 2 — Perception of knowledge in the system. (GIF 5992 kb) [file 42492_2019_19_MOESM2_ESM.gif]
